# *catena*-Poly[[bis­(nitrato-κ^2^ *O*,*O*′)zinc(II)]-μ-4,4′-bis­(pyrazol-1-ylmeth­yl)biphenyl-κ^2^ *N* ^2^:*N* ^2′^]

**DOI:** 10.1107/S1600536810018738

**Published:** 2010-05-22

**Authors:** Xue Wang, Jin-Sheng Gao, Zhi-Yong Ding, Guang-Feng Hou

**Affiliations:** a College of Chemistry and Materials Science, Heilongjiang University, Harbin 150080, People’s Republic of China; b Engineering Research Center of Pesticide of Heilongjiang Province, Heilongjiang University, Harbin 150080, People’s Republic of China; c Daqing New Century Industrial Co. Ltd, Daqing 163511, People’s Republic of China

## Abstract

In the title compound, [Zn(NO_3_)_2_(C_20_H_18_N_4_)]_*n*_, the Zn^II^ atom lies on a crystallographic twofold axis and the coordination geometry can be considered as a slightly distorted tetra­hedron defined by two O atoms from two nitrate groups and two N atoms from two ligand mol­ecules. A distorted octa­hedron may be assumed when two of the symmetry-related nitrate O atoms, with Zn—O distances of 2.528 (2) Å, are added to the coordination environment. Another twofold axis, passing through the middle of the biphenyl bonds, is observed in the crystal structure. A chain along [101] is built up by the ligands linking the Zn^II^ ions.

## Related literature

For a related polymeric bis­(pyrazole) dinitratocobalt(II) structure, see: Chen *et al.* (1997 ▶). For the synthesis and structure of a three-dimensional polymeric Zn(II) network compound, see: Zhu *et al.* (2002 ▶).
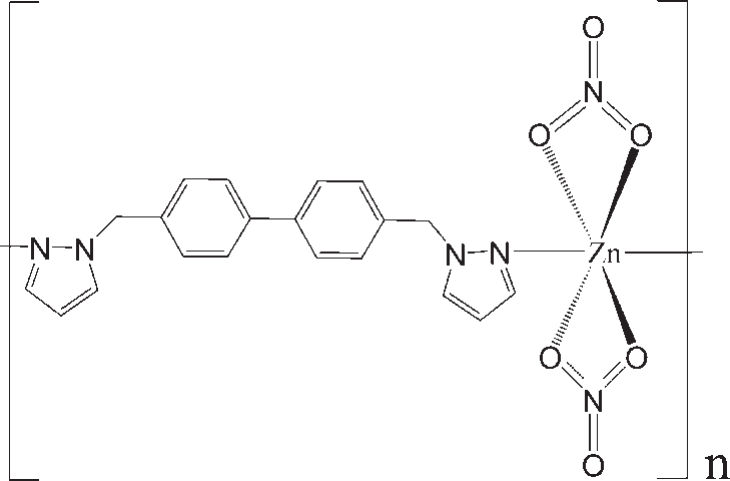


## Experimental

### 

#### Crystal data

[Zn(NO_3_)_2_(C_20_H_18_N_4_)]
*M* *_r_* = 503.77
Monoclinic, 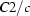

*a* = 14.088 (3) Å
*b* = 13.780 (3) Å
*c* = 10.744 (2) Å
β = 95.76 (3)°
*V* = 2075.2 (7) Å^3^
*Z* = 4
Mo *K*α radiation
μ = 1.24 mm^−1^
*T* = 291 K
0.47 × 0.31 × 0.27 mm

#### Data collection

Rigaku R-AXIS RAPID diffractometer
Absorption correction: multi-scan (*ABSCOR*; Higashi, 1995 ▶) *T*_min_ = 0.595, *T*_max_ = 0.728
9881 measured reflections
2361 independent reflections
2107 reflections with *I* > 2σ(*I*)
*R*_int_ = 0.027

#### Refinement

*R*[*F* ^2^ > 2σ(*F* ^2^)] = 0.029
*wR*(*F* ^2^) = 0.072
*S* = 1.07
2361 reflections
150 parameters
H-atom parameters constrained
Δρ_max_ = 0.29 e Å^−3^
Δρ_min_ = −0.30 e Å^−3^

### 

Data collection: *RAPID-AUTO* (Rigaku, 1998 ▶); cell refinement: *RAPID-AUTO*; data reduction: *CrystalStructure* (Rigaku/MSC, 2002 ▶); program(s) used to solve structure: *SHELXS97* (Sheldrick, 2008 ▶); program(s) used to refine structure: *SHELXL97* (Sheldrick, 2008 ▶); molecular graphics: *SHELXTL* (Sheldrick, 2008 ▶); software used to prepare material for publication: *SHELXL97*.

## Supplementary Material

Crystal structure: contains datablocks I, global. DOI: 10.1107/S1600536810018738/si2259sup1.cif

Structure factors: contains datablocks I. DOI: 10.1107/S1600536810018738/si2259Isup2.hkl

Additional supplementary materials:  crystallographic information; 3D view; checkCIF report

## Figures and Tables

**Table 1:** Selected bond lengths (Å)

N1—Zn1	2.0240 (14)
O1—Zn1	2.0169 (14)
O2—Zn1	2.5277 (16)

**Fig. 1.**
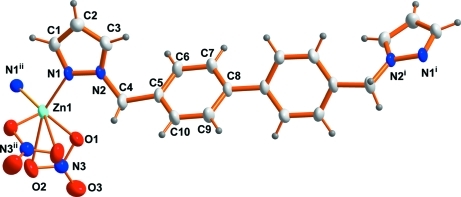
The molecular structure of the title compound, showing the atom-labeling scheme and displacement ellipsoids drawn at the 50% probability level. symmetry codes: i = -x + 1, y, -z - 0.5; ii = -x + 2, y, -z + 0.5

**Fig. 2.**
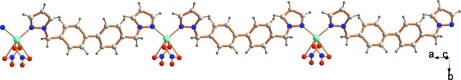
A view of the one dimensional structure of the title compound extending along the [1 0 1] direction.

## supplementary crystallographic information

### Comment

The structures of the metal derivative 1,4-bis(pyrazole) benzene are known for zinc and cobalt (Chen *et al.*, 1997). In order to enrich the research of this kinds of ligand, a new ligand 4,4'-bis(pyrazole) biphenyl with longer spacer was synthesized, and which are used in the preparation of coordination compound with zinc dinitrate.

The central Zn atom lies on a crystallographic twofold axis and the coordination geometry can be considered as a slightly distorted tetrahedron defined by two O atoms from two nitrate groups and two N atoms from two ligand molecules. A distorted octahedron may be assumed when two of the C_2_ related nitrate oxygen atoms with Zn—O distances of 2.528 (2) Å are added (Table 1) to the coordination environment. Another twofold axis, passing through the middle of the biphenyl bonds, is observed in the crystal structure (Figure 1).

A one dimensional chain is built up by the ligands linking the Zn^II^ ions along the [1 0 1] direction (Figure 2).

### Experimental

The 4,4'-bis(pyrazole-1-ylmethyl) biphenyl was synthesized by the reaction of pyrazole and 4,4'-bis(chloro) bibenzene under alkaline condition (Zhu *et al.*, 2002). Zinc(II) dinitrate hexahydrate (0.595 g, 2 mmol) and 4,4'-bis(pyrazole-1-ylmethyl) biphenyl (0.618 g, 2 mmol) were dissolved in ethanol (20 ml), colorless block-shaped crystals of the title compound were obtained by slow evaporation of ethanol solution after several days.

### Refinement

H atoms bound to C atoms were placed in calculated positions and treated as riding on their parent atoms, with C—H = 0.93 Å (aromatic), C—H = 0.97 Å (methylene), and with *U*_iso_(H) = 1.2*U*_eq_(C).

### Crystal data

**Table:**

[Zn(NO_3_)_2_(C_20_H_18_N_4_)]	*F*(000) = 1032
*M**_r_* = 503.77	*D*_x_ = 1.612 Mg m^−^^3^
Monoclinic, *C*2/*c*	Mo *K*α radiation, λ = 0.71073 Å
Hall symbol: -C 2yc	Cell parameters from 8577 reflections
*a* = 14.088 (3) Å	θ = 3.5–27.5°
*b* = 13.780 (3) Å	µ = 1.24 mm^−^^1^
*c* = 10.744 (2) Å	*T* = 291 K
β = 95.76 (3)°	Block, colorless
*V* = 2075.2 (7) Å^3^	0.47 × 0.31 × 0.27 mm
*Z* = 4	

### Data collection

**Table:**

Rigaku R-AXIS RAPID diffractometer	2361 independent reflections
Radiation source: fine-focus sealed tube	2107 reflections with *I* > 2σ(*I*)
graphite	*R*_int_ = 0.027
ω scan	θ_max_ = 27.5°, θ_min_ = 3.5°
Absorption correction: multi-scan (*ABSCOR*; Higashi, 1995)	*h* = −18→18
*T*_min_ = 0.595, *T*_max_ = 0.728	*k* = −17→17
9881 measured reflections	*l* = −13→12

### Refinement

**Table:**

Refinement on *F*^2^	Primary atom site location: structure-invariant direct methods
Least-squares matrix: full	Secondary atom site location: difference Fourier map
*R*[*F*^2^ > 2σ(*F*^2^)] = 0.029	Hydrogen site location: inferred from neighbouring sites
*wR*(*F*^2^) = 0.072	H-atom parameters constrained
*S* = 1.07	*w* = 1/[σ^2^(*F*_o_^2^) + (0.0366*P*)^2^ + 1.1611*P*] where *P* = (*F*_o_^2^ + 2*F*_c_^2^)/3
2361 reflections	(Δ/σ)_max_ = 0.001
150 parameters	Δρ_max_ = 0.28 e Å^−^^3^
0 restraints	Δρ_min_ = −0.30 e Å^−^^3^

### Special details

**Table:**

Geometry. All esds (except the esd in the dihedral angle between two l.s. planes) are estimated using the full covariance matrix. The cell esds are taken into account individually in the estimation of esds in distances, angles and torsion angles; correlations between esds in cell parameters are only used when they are defined by crystal symmetry. An approximate (isotropic) treatment of cell esds is used for estimating esds involving l.s. planes.
Refinement. Refinement of *F*^2^ against ALL reflections. The weighted *R*-factor wR and goodness of fit *S* are based on *F*^2^, conventional *R*-factors *R* are based on *F*, with *F* set to zero for negative *F*^2^. The threshold expression of *F*^2^ > σ(*F*^2^) is used only for calculating *R*-factors(gt) etc. and is not relevant to the choice of reflections for refinement. *R*-factors based on *F*^2^ are statistically about twice as large as those based on *F*, and *R*- factors based on ALL data will be even larger.

### Fractional atomic coordinates and isotropic or equivalent isotropic displacement parameters (Å^2^)

**Table:**

	*x*	*y*	*z*	*U*_iso_*/*U*_eq_	
C1	0.88226 (13)	0.85458 (13)	0.30265 (17)	0.0400 (4)	
H1	0.9299	0.8328	0.3623	0.048*	
C2	0.79425 (13)	0.81078 (13)	0.27656 (17)	0.0422 (4)	
H2	0.7719	0.7555	0.3138	0.051*	
C3	0.74719 (12)	0.86596 (12)	0.18459 (17)	0.0378 (4)	
H3	0.6857	0.8550	0.1472	0.045*	
C4	0.79018 (13)	1.01784 (13)	0.06773 (19)	0.0448 (5)	
H4A	0.8446	1.0209	0.0192	0.054*	
H4B	0.7877	1.0785	0.1132	0.054*	
C5	0.70008 (12)	1.00840 (13)	−0.02129 (16)	0.0344 (4)	
C6	0.68292 (12)	0.92829 (13)	−0.09799 (17)	0.0394 (4)	
H6	0.7250	0.8761	−0.0910	0.047*	
C7	0.60321 (12)	0.92531 (13)	−0.18549 (16)	0.0371 (4)	
H7	0.5913	0.8701	−0.2345	0.045*	
C8	0.54112 (11)	1.00366 (12)	−0.20065 (15)	0.0304 (3)	
C9	0.55789 (12)	1.08310 (12)	−0.12237 (16)	0.0379 (4)	
H9	0.5164	1.1357	−0.1299	0.045*	
C10	0.63608 (12)	1.08471 (13)	−0.03278 (16)	0.0392 (4)	
H10	0.6456	1.1379	0.0203	0.047*	
N1	0.88943 (9)	0.93271 (10)	0.23026 (13)	0.0336 (3)	
N2	0.80483 (9)	0.93853 (10)	0.15759 (12)	0.0307 (3)	
N3	1.06552 (11)	1.16224 (10)	0.10426 (14)	0.0389 (3)	
O1	1.00867 (10)	1.09113 (9)	0.08271 (13)	0.0476 (3)	
O2	1.10427 (11)	1.17141 (11)	0.21293 (14)	0.0588 (4)	
O3	1.08004 (13)	1.21789 (11)	0.02032 (15)	0.0678 (5)	
Zn1	1.0000	1.027091 (19)	0.2500	0.03491 (11)	

### Atomic displacement parameters (Å^2^)

**Table:**

	*U*^11^	*U*^22^	*U*^33^	*U*^12^	*U*^13^	*U*^23^
C1	0.0374 (9)	0.0386 (9)	0.0404 (9)	0.0011 (7)	−0.0139 (7)	0.0046 (8)
C2	0.0406 (10)	0.0393 (9)	0.0449 (10)	−0.0062 (7)	−0.0043 (8)	0.0060 (8)
C3	0.0263 (8)	0.0419 (9)	0.0430 (9)	−0.0074 (7)	−0.0070 (7)	−0.0014 (8)
C4	0.0330 (9)	0.0453 (10)	0.0506 (11)	−0.0088 (7)	−0.0235 (8)	0.0106 (8)
C5	0.0252 (8)	0.0407 (8)	0.0342 (9)	−0.0030 (6)	−0.0121 (6)	0.0055 (7)
C6	0.0300 (8)	0.0422 (9)	0.0424 (9)	0.0094 (7)	−0.0135 (7)	−0.0017 (8)
C7	0.0343 (9)	0.0380 (9)	0.0359 (8)	0.0042 (7)	−0.0123 (7)	−0.0071 (7)
C8	0.0227 (8)	0.0379 (8)	0.0285 (8)	−0.0009 (6)	−0.0080 (6)	0.0003 (6)
C9	0.0301 (8)	0.0374 (9)	0.0428 (9)	0.0065 (7)	−0.0122 (7)	−0.0038 (7)
C10	0.0372 (9)	0.0378 (9)	0.0386 (9)	−0.0014 (7)	−0.0151 (7)	−0.0064 (7)
N1	0.0238 (6)	0.0356 (7)	0.0377 (7)	−0.0008 (5)	−0.0151 (5)	0.0003 (6)
N2	0.0214 (6)	0.0342 (6)	0.0337 (7)	−0.0018 (5)	−0.0113 (5)	−0.0008 (6)
N3	0.0339 (8)	0.0359 (7)	0.0456 (8)	−0.0004 (6)	−0.0022 (6)	0.0005 (7)
O1	0.0461 (8)	0.0409 (7)	0.0521 (8)	−0.0107 (6)	−0.0130 (6)	0.0012 (6)
O2	0.0503 (9)	0.0668 (9)	0.0550 (8)	−0.0159 (7)	−0.0154 (7)	−0.0043 (7)
O3	0.0858 (12)	0.0570 (9)	0.0600 (9)	−0.0229 (9)	0.0047 (8)	0.0089 (8)
Zn1	0.02434 (15)	0.03443 (16)	0.04240 (18)	0.000	−0.01422 (11)	0.000

### Geometric parameters (Å, °)

**Table:**

C1—N1	1.338 (2)	C7—H7	0.9300
C1—C2	1.382 (2)	C8—C9	1.386 (2)
C1—H1	0.9300	C8—C8^i^	1.490 (3)
C2—C3	1.365 (2)	C9—C10	1.388 (2)
C2—H2	0.9300	C9—H9	0.9300
C3—N2	1.338 (2)	C10—H10	0.9300
C3—H3	0.9300	N1—N2	1.3602 (17)
C4—N2	1.459 (2)	N1—Zn1	2.0240 (14)
C4—C5	1.516 (2)	N3—O3	1.217 (2)
C4—H4A	0.9700	N3—O2	1.245 (2)
C4—H4B	0.9700	N3—O1	1.2721 (19)
C5—C10	1.383 (2)	O1—Zn1	2.0169 (14)
C5—C6	1.384 (3)	O2—Zn1	2.5277 (16)
C6—C7	1.391 (2)	Zn1—O1^ii^	2.0169 (14)
C6—H6	0.9300	Zn1—N1^ii^	2.0240 (14)
C7—C8	1.389 (2)		
			
N1—C1—C2	110.62 (15)	C8—C9—C10	120.55 (15)
N1—C1—H1	124.7	C8—C9—H9	119.7
C2—C1—H1	124.7	C10—C9—H9	119.7
C3—C2—C1	105.32 (16)	C5—C10—C9	120.98 (16)
C3—C2—H2	127.3	C5—C10—H10	119.5
C1—C2—H2	127.3	C9—C10—H10	119.5
N2—C3—C2	108.18 (15)	C1—N1—N2	105.55 (13)
N2—C3—H3	125.9	C1—N1—Zn1	123.95 (11)
C2—C3—H3	125.9	N2—N1—Zn1	130.02 (11)
N2—C4—C5	114.01 (14)	C3—N2—N1	110.32 (13)
N2—C4—H4A	108.8	C3—N2—C4	130.89 (13)
C5—C4—H4A	108.8	N1—N2—C4	118.79 (13)
N2—C4—H4B	108.8	O3—N3—O2	122.73 (16)
C5—C4—H4B	108.8	O3—N3—O1	120.02 (16)
H4A—C4—H4B	107.6	O2—N3—O1	117.25 (15)
C10—C5—C6	118.68 (14)	N3—O1—Zn1	105.56 (10)
C10—C5—C4	119.23 (16)	N3—O2—Zn1	81.99 (10)
C6—C5—C4	121.94 (16)	O1^ii^—Zn1—O1	128.11 (8)
C5—C6—C7	120.45 (15)	O1^ii^—Zn1—N1	105.05 (6)
C5—C6—H6	119.8	O1—Zn1—N1	107.61 (6)
C7—C6—H6	119.8	O1^ii^—Zn1—N1^ii^	107.61 (6)
C8—C7—C6	120.83 (15)	O1—Zn1—N1^ii^	105.05 (6)
C8—C7—H7	119.6	N1—Zn1—N1^ii^	100.03 (8)
C6—C7—H7	119.6	O1^ii^—Zn1—O2	83.36 (6)
C9—C8—C7	118.41 (14)	O1—Zn1—O2	55.06 (5)
C9—C8—C8^i^	120.32 (11)	N1—Zn1—O2	160.46 (5)
C7—C8—C8^i^	121.27 (11)	N1^ii^—Zn1—O2	93.94 (6)

Symmetry codes: (i) −*x*+1, *y*, −*z*−1/2; (ii) −*x*+2, *y*, −*z*+1/2.
